# Gaming Technology for Pediatric Neurorehabilitation: A Systematic Review

**DOI:** 10.3389/fped.2022.775356

**Published:** 2022-01-28

**Authors:** Marco Iosa, Cristiano Maria Verrelli, Amalia Egle Gentile, Martino Ruggieri, Agata Polizzi

**Affiliations:** ^1^Department of Psychology, Sapienza University of Rome, Rome, Italy; ^2^Scientific Institute for Research, Hospitalization and Healthcare (IRCCS) Santa Lucia Foundation, Rome, Italy; ^3^Electronic Engineering Department, University of Rome Tor Vergata, Rome, Italy; ^4^National Centre for Rare Diseases, Istituto Superiore di Sanità, Rome, Italy; ^5^Unit of Rare Diseases of the Nervous System in Childhood, Department of Clinical and Experimental Medicine, University of Catania, Catania, Italy; ^6^Department of Educational Science, Chair of Pediatrics, University of Catania, Catania, Italy

**Keywords:** children, adolescents, neurorehabilitation, videogames, virtual reality, exergaming

## Abstract

**Introduction:**

The emergence of gaming technologies, such as videogames and virtual reality, provides a wide variety of possibilities in intensively and enjoyably performing rehabilitation for children with neurological disorders. Solid evidence-based results are however required to promote the use of different gaming technologies in pediatric neurorehabilitation, while simultaneously exploring new related directions concerning neuro-monitoring and rehabilitation in familiar settings.

**Aim of the Study and Methods:**

In order to analyze the state of the art regarding the available gaming technologies for pediatric neurorehabilitation, Scopus and Pubmed Databases have been searched by following: PRISMA statements, PICOs classification, and PEDro scoring.

**Results:**

43 studies have been collected and classified as follows: 11 feasibility studies; six studies proposing home-system solutions; nine studies presenting gamified robotic devices; nine longitudinal intervention trials; and eight reviews. Most of them rely on feasibility or pilot trials characterized by small sample sizes and short durations; different methodologies, outcome assessments and terminologies are involved; the explored spectrum of neurological conditions turns out to be scanty, mainly including the most common and wider debilitating groups of conditions in pediatric neurology: cerebral palsy, brain injuries and autism.

**Conclusion:**

Even though it highlights reduced possibilities of drawing evidence-based conclusions due to the above outlined biases, this systematic review raises awareness among pediatricians and other health professionals about gaming technologies. Such a review also points out a definite need of rigorous studies that clearly refer to the underlying neuroscientific principles.

## Introduction

Neurorehabilitation (or neurological rehabilitation) is a multi-professional physician-led approach to healthcare aiming at reducing disability and at improving functions affected by damaged nervous system (1). Neurorehabilitation is often regarded as a long, stressful, and unexciting treatment, especially in children affected by cerebral palsy, acquired brain injury, developmental dyspraxia, or other severely debilitating neurological impairments. One way of dealing with—and bypass—repetitive and dull interventions is to include some elements of play, by creating games to boost motivation during treatment procedures, so as to reduce stress and favor compliance (1).

Effective neurorehabilitation conforms to the sensorimotor and cognitive learning model, whose main principles are engagement as well as task-oriented and intensive practice. All those aspects require strong devotion, often difficult to pursuit in children. In respect with this, the use of emerging gaming technology, designed to be funny and enjoyable, would allow children to perform an intensive and prolonged repetition of the body movements requested to gain high scores in the game. No lack of interest arises when reinforcement and feedbacks are received, which in turn are relevant tips in motor learning for enhancing neuroplasticity (2–4).

Even though motivation might be increased in technologically assisted neurorehabilitation with no task gamification [e.g., by using art in virtual reality protocols (5)], gaming seems to be the most simple and attractive solution for children to enhance their active participation to rehabilitation.

Despite the bulk of clinical evidence regarding the use of serious exergames in children is rather insufficient, currently there is a continuous and progressive spread of gaming technology in neurorehabilitation. Pediatricians should be aware of such a new intensive and enjoyable approach, showing promising results for an expanding target of diseases of the nervous system. Several studies and some reviews have dealt with the use of videogames in pediatric neurorehabilitation, but our focus on gaming technology allows us to include software and hardware prototypes, virtual reality, as well as computerized exergames used in tele- and robotic-rehabilitation.

This review aims at analyzing the state of the art regarding the available gaming technologies for pediatric neurorehabilitation, by analyzing the evidences of their efficacy, when differentiated among products, applications, and combinations with other technologies.

## Materials and Methods

**Figure 1:**
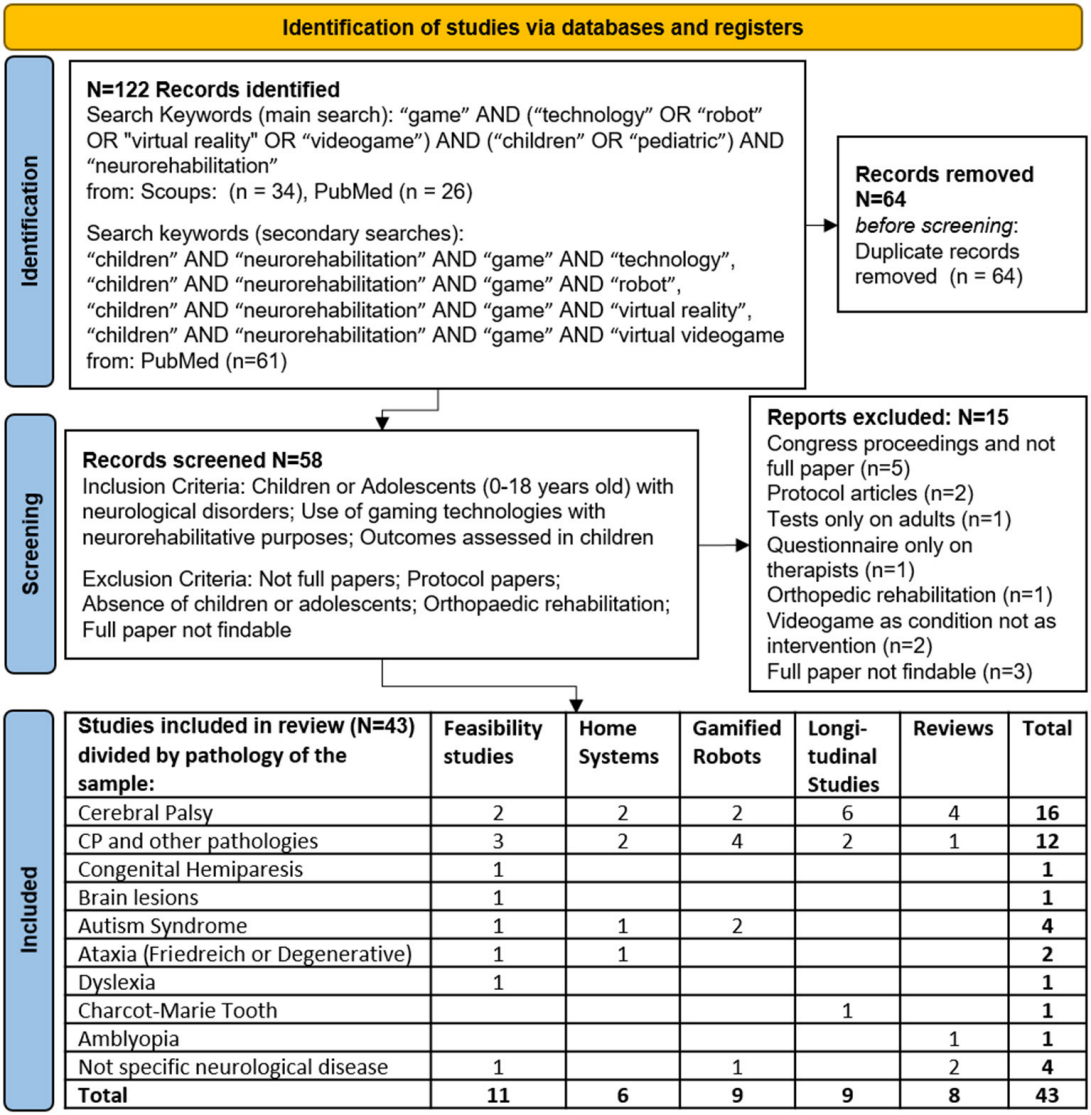
Flow diagram of the mini review described according to the PRISMA statement 2020.

A systematic search was carried out according to the PRISMA criteria (6) on the 21st of June, 2021, without date limits (see Figure 1).

The search was conducted on both PubMed and Scopus databases. In the primary search, the following keywords were used: “game” AND (“technology” OR “robot” OR “virtual reality” OR “videogame”) AND (“children” OR “pediatric”) AND “neurorehabilitation”. In a secondary adjunctive search, conducted solely on PubMed the following combinations of keywords were used in adjunction (AND) to “children” AND “neurorehabilitation”: “game” AND “technology”, “game” AND “robot”, “game” AND “virtual reality”, “game” AND “virtual videogame”.

After duplication removal, papers were screened according to the following Inclusion/Exclusion criteria. Inclusion criteria were: Children or Adolescents (0–18 years old) with neurological disorders; Use of gaming technologies with neurorehabilitative purposes; Outcomes assessed in children. Exclusion criteria were: not full papers; protocol papers; absence of children or adolescents; orthopedic rehabilitation; full paper not findable.

Two researchers worked independently. The findings were then merged together. Two additional researchers verified the whole process execution, with the aim of confirming or questioning the adequacy of the obtained sources. All the researchers worked together for the final assessment. No automation tools were used in this review.

Longitudinal studies were reported by using PICOS-criteria and their quality was assessed *via* the PEDro-scale (7).

## Results

The results of the present search strategy are reported in Figure 1.

### Feasibility Studies: Commercial Consoles and *ad-hoc* Gaming Technology

Eleven articles (8–18) concerned feasibility studies on gaming technologies in pediatric neurorehabilitation. Most of them were based on a single session experiment.

Evidence promoting the usability and utility of such devices was supported by comparative results concerning the performance of children with neurological diseases and children with typical development during or after a single videogame-based neurorehabilitation session involving commercial consoles (Nintendo Wii and Microsoft Xbox Kinect) (8–10) or more sophisticated technologies (11). It is worth mentioning that, among the commercial consoles, Microsoft Kinect was the most used, due to the feasibility of total body tracking and recording during games (8, 12).

Other studies were based on hardware and software developed *ad-hoc* for neurorehabilitation. In particular, Kommalapati and Michmizos (13) developed a videogame in which the child's movements were recorded and applied to an avatar viewable in 3rd person, with the idea that visual observation of one's own movements is able to activate the “mirror neuron system,” favoring sensorimotor learning. Bortone et al. (14, 15) combined the use of videogames with a wearable haptic interface: significant differences were found in kinematics parameters recorded in a single session among children with cerebral palsy (CP), developmental dyspraxia, typical development, and also healthy adults. The importance of these wearable devices for differentiating between physiological and compensatory movements was demonstrated also in another study using the YouGrabber^®^ on 33 children with brain lesions (16).

Finally, the use of gaming technology was also used to perform cognitive neurorehabilitation, such as for the emotional expression re-education of children with autism spectrum disorders (ASD) (17) and the improvement of reading abilities in 10 children with dyslexia (by using Nintendo-Wii) (18), though the latter study did not report any significant change in the investigated sample (18).

### Gaming Technologies in Home Neurorehabilitation

Six studies (19–24) proposed the use of home-based computer-enhanced therapy for children with early-onset ataxias (19), CP (20–22), ASD (23) and in general for children with neurological disorders (24).

Summa et al. (19) proposed the SaraHome system, which combines a Kinect and a Leap Motion Controller with an *ad-hoc* software: standardized motor tasks performed by 10 children affected by early onset ataxias under the caregivers' assistance were acquired. NExT (Neuroplasticity-trained-EXercise-Trainer) was another system based on an egg-shaped controller for videogaming, whose usability was positively tested in children with CP (20). Similar results were obtained for the Timocco, used at home in a case report on a child with CP (21).

Gerber et al. (22) tested the usability of a portable version of the YouGrabber^®^ system for hand and arm training at home in 15 children with CP. However, the system was error prone and the requested support exceeded the one that could be provided by clinical therapists. A similar observation, concerning the errors related to a specifically developed software was done by Kang and Chang (23), who used a Kinect for training 6 children with ASD to take a shower independently. Valdés et al. (24) stated that also some other aspects should have been taken into account for improving usability and efficacy of home-based systems: ability to track compensatory movements, clinical considerations in game selection, the provision of kinematic and treatment progress reports to participants, and effective communication and training of therapists and participants.

### Gaming and Robots in Pediatric Neurorehabilitation

Nine studies were focused on pediatric robotic rehabilitation using gaming technology (25–33). A simple mechanized manipulandum, moved by children with CP to interact with the videogame, was used in the ROBiGAME project (25). The kinematic and kinetic parameters recorded by the robot were proved to be significantly correlated with the clinical assessment. Differently, the robot could act as an exoskeleton guiding the upper limb(s) of children such as doing by ChARMin (26) or Armeo-Spring (27) robots, with the latter showing a significant improvement as compared to baseline in three adolescents with CP after three daily 70 min-long sessions. Also the Lokomat, a robot for gait training, was used in combination with videogames in children with neurological gait disorders, finding that the amount of activity was increased in dual-task exercises (28) and was related to the demanding level of gaming (29). The effects on active participation to a robotic gait training combined with a soccer videogame was found to be similar to the one obtained in the presence of verbal feedbacks of therapists (30).

In three studies, a mobile toy robot was tested during its interaction with children (31–33). Four children with ASD during spontaneous game showed significant interaction with the robot (31), with positive emotions arising during the subsequent social interactions with other people (32). Similar results were obtained by using RoboCog, with significant interactions between pediatric patients and robot resulting, but these interactions depended on the level of attention and collaborative attitudes of children (33).

### Gaming Technologies in Longitudinal Neurorehabilitation Trials

Our search identified nine longitudinal studies as reported in Table 1 (34–42). According to the PEDro scores, one of the most interesting paper was the one by Choi et al. (35), showing that children with chronic brain injury (including CP), treated by virtual reality and occupational therapy, significantly improved upper-limb dexterity functions, performance of daily activities, and forearm supination, as compared to the control group. Wider improvements were observed for children with more severe motor impairment. Gorelik et al. (38) showed that the Krisaf training simulator-based rehabilitation led to significant refinement of motor capabilities in children with spastic CP. Also, Tarakci et al. (39) reported a significant balance improvement in children and adolescents with CP treated by using Nintendo-Wii-Fit balance board. Progress in balance, after neurorehabilitation with Xbox and Kinect, was also reported in a child with Charcot-Marie-Tooth disease (41).

**Table 1:** The longitudinal studies entered in this review.

**References**	**Children disease (sample)**	**Mean age ±s.d. (years)**	**Type of study intervention and comparison (type and time)**	**Outcome**	**PEDro score (max 9)**
Bortone et al. (34)	CP (*N* = 3) DD (*N* = 5)	10.13 ± 2.59	Single-blind randomized controlled crossoverVR with wearable haptic feedback vs. conventional therapy 2 sessions per week for 4 weks	GMFCS and MACS for CP, ZP for DD	8
Choi et al. (35)	BI (*N* = 80)	5.8 ± 2.1	RCT VR (30 min) for upper limb plus occupational therapy (30 min) vs. conventional occupational therapy; 20 sessions over 4 weeks	MA-2; PEDICAT; ULPRS; computerized 3D motion analysis	8
Decavele et al. (36)	CP (*N* = 32)	10	Crossover RCT Physiotherapy and videogame combined with Kinect and Wii balance board vs. physiotherapy alone 45 min sessions, twice a week, 12 weeks	GAS; TCMS; PBS; DMQ	7
Do et al. (10)	CP (*N* = 3)	6 ± 1	ABA designed study Nintendo Wii game Baseline (4 sessions); Videogame (12 sessions); follow-up (4 sessions), 30 min each session	WMFT; PMAL	4
Golomb et al. (37)	CP (*N* = 1)	15	Case Report 5DT 5 Ultra Glove and PlayStation3; 14 months and 14 months of follow-up	Grip strength; Jebsen test of hand function	NA
Gorelik et al. (38)	CP (*N* = 16)	8	RCT VR Krisaf training simulator vs. physiotherapy 2 sessions of 40 min per week for 8 months.	Time of holding a postural position	5
Tarakci et al. (39)	CP (*N* = 30)	10.46 ± 2.69	RCT NDT and Nintendo Wii-Fit Balance Games vs. conventional balance training 24 sessions of 50 min; 2 sessions per week	Functional Reach Test; Sit-to-Stand Test; Timed Get up and Go Test.	
Zoccolillo et al. (40)	CP (*N* = 22)	6.89 ± 1.91	Cross-over RCT X-box Kinect videogames vs. conventional therapy 2 sessions of 30 min per week for 8 weeks	QUEST; Abilhand-kids	7

*BI, Brain injury; CP, cerebral palsy; DD, developmental dyspraxia; TD, typical development; NDT, Neurodevelopmental treatment; RCT, Randomized controlled trial; VR, Virtual Reality; NA, not assessed; Assessment Tools: DMQ, Dimensions of Mastery Motivation Questionnaire; GAS, Goal Attainment Scale; GMFCS, Gross Motor Function Classification System; MACS, Manual Ability Classification System; MA-2, Melbourne Assessment of Unilateral Upper Limb Function-2; PBS, Pediatric Balance Scale; PEDICAT, Pediatric Evaluation of Disability Inventory Computer Adaptive Test; PMAL, Pediatric Motor Activity Log; QUEST, Quality of Upper Extremity Skills Test; TCMS, Trunk Control Measurement Scale; ULPRS, Upper Limb Physician's Rating Scale; WMFT, Wolf Motor Function Test; (WMFT); ZP, Zoia's protocol*.

More specific results were found by Zoccolillo et al. (40): children with CP had a higher gain in the quality of upper limb extremity skills when treated by using videogames, and higher improvement in manual activities when treated with conventional therapy. The authors justified their results in light of the used commercial console, the X-box with Microsoft-Kinect, that records gross movements of limbs and not fine movements of the fingers.

Bortone and colleagues (34) performed a cross-over randomized controlled trial using the same system that was positively tested for feasibility in their previous studies (14, 15) combining an immersive virtual environment with a wearable haptic device. No statistically significant differences were found with respect to conventional therapy, and the authors interpreted this result as a non-inferiority of gaming technology vs. conventional therapy in increasing upper extremity function in children with neuromotor deficits.

The study of de Paula et al. (42) was also fascinating:, children with CP were able (i) to ameliorate their performance by using a videogame on a smartphone; (ii) to maintain the acquired skill in a retention test; (iii) to transfer the skill to a similar, but different, virtual task.

A controversial issue was the long-term effects of gaming technology in neurorehabilitation. Decavele et al. (36) showed that a combined approach of conventional physiotherapy and videogames specifically designed for rehabilitation showed significant effects on individually defined therapy goals, dynamic sitting balance, and standing exercises. However, these differences were lost at the 3-month follow-up, so that the authors suggested that, given this lack of persistent effect, a continuous individual goal-oriented physiotherapy with the addition of gaming was needed. Different results were reported in a case-study of an adolescent with hemiplegic CP by using a home-based telerehabilitation system incorporating a 5DT 5-Ultra-Glove to interact with PlayStation3 game console programmed with custom rehabilitation games: the improvements obtained with 14 months of gaming were maintained after 14 months of follow-up (37).

### Findings in Review Studies

Ravi et al. (43), in their review on the use of videogames in children and adolescents with CP, reported moderate evidence for balance improvement and overall motor development, albeit still limited results for other motor skills. These results were confirmed by a meta-analysis reporting that videogames played a positive role in the improvement of balance of children with CP, despite authors claimed caution for methodological defects (e.g., difference in measurement, heterogeneity of control groups, intervention combined with other treatments, etc.) (44). Bonnechére et al. (45) also highlighted the difficulties in comparing the studies because of the lack of standardization in rehabilitation strategies and used outcomes, limiting the possibility to provide solid evidence-based conclusions. They and other authors (46) claimed the need of standardizing the protocols to improve treatment comparisons.

Lai et al. (47) analyzed the positive effects of leisure-time physical activity in children and adults with CP, reporting improvements in health, fitness, and physical functions attained by interventions including exercise training, active videogames, recreation activities, behavioral coaching, and motor skills training, with telehealth technology, and community resources.

Jurdi et al. (2) showed that the most common approach for the use of gaming technology in pediatric hospitals is to use mono-user games with traditional computers or monitor-based video consoles, which serve as a distractor for fearful interventions or a motivator for physical rehabilitation. Interestingly, they suggested to include, in the gaming approach, some features for favoring socialization, coping with emotions, or fostering physical mobility.

Coco Martin et al. (48) analyzed the use of VR for inducing neuroplasticity in children with amblyopia. They reported that head-mounted displays are mostly well-tolerated by patients during short exposures and do not cause significant long-term side effects, although their use has been occasionally associated with some visual discomfort and other complications in certain types of subjects. They concluded that a larger number of studies is needed to confirm these promising therapies in controlled randomized clinical trials.

Deutsch et al. analyzed the energy consumption during videogaming with commercial consoles, finding that adults and children with mild severe forms of CP played the videogames at vigorous levels, whereas those with severe CP played them at low levels, concluding that videogames could be useful for wellness promotion (46).

Jannsen and colleagues wrote a perspective study for highlighting how the gamification of therapy has the potential to increase participants' motivation and engagement in therapy, owing to the involvement of reward-related dopaminergic systems in the brain that are known to facilitate learning through long-term potentiation of neural connections (49).

## Discussion

The literature analysis revealed that most articles regarding the available gaming technologies for neurorehabilitation and their use in pediatric patients belong to feasibility or pilot studies with small sample sizes, few sessions (often only one), varied methodologies and outcome measures, and without clear neuroscientific principle behind the videogame setup. Home prototypes often required therapist assistance to avoid errors, compensatory strategies and to guide the game selection. Gaming technology seemed to be helpful in robotic therapy for motivating children during their training, as of both motor and cognitive functions. Only a few studies represented high-quality randomized controlled trials. The most used game tools are commercial consoles with games not specifically developed to neurorehabilitation. This could be due to the fact that commercial devices are more robust and more attractive than the prototypes developed for research (22). However, it must be noted that encouraging evidence stems from games specifically developed for monitoring and for rehabilitation purposes, to be applied to face neurological diseases in household settings: these devices are specifically built up in order to avoid interferences with the daily activities of patients and to attain clear advantages for both patients and caregivers (50).

There are also some questionable aspects that are reported in the reviews: e.g., the absence of statistically significant differences interpreted as a proof of non-inferiority of gaming technologies with respect to conventional therapy (34, 36), the improper use of the expression “virtual reality” for videogames (8, 10, 29, 43), the different approaches and methodologies that lowered the possibility of comparisons among studies (45, 46).

The use of gaming for neurorehabilitation purposes was mainly motivated by the increment of engagement and reduction of stress and boredom. The selection of games itself was mainly related to the movements to be performed and on how much enjoyable and entertaining was the game. Just a few studies reported on a clear neuroscientific principle behind the design of the game. Kommalapati and Michmizos (13) clearly referred to structural (mirror neurons) and functional (action-observation learning) neuroscientific knowledge; Jannsen et al. (49) described the involvement of reward-related dopaminergic systems during videogaming. Zoccolillo et al. linked the characteristics of the commercial devices with the specific observed outcomes (40).

A further important issue emerging from the present study is that the most common clinical application areas of gaming technology so far included cerebral palsy. It leads to have more evidences about the efficacy of gaming technologies in rehabilitation of a static injury (such as cerebral palsy) than in other conditions such as traumatic brain injuries or genetic diseases. About it, there is a number of application areas of pediatric neurology hardly addressed in the reviewed studies, such as for example rare diseases (RDs), which are complex and heterogeneous chronic conditions, as yet including almost 10,000 recognized disorders affecting >300,000,000 persons in the world (51), often resulting in various degrees of neurological impairment (52). For about 95% of such chronic conditions, neurorehabilitation is a compelling long-life supportive treatment (53): in this respect, emerging technologies could provide more precise data from video-analyses or wearable sensors, including accelerometer, gyroscope, magnetometer, quaternion and barometer-synced data (54). These tools might even allow for, in the exergames context, the instrumental evaluation of children's motor abilities through the latest outcome measures capturing the level of distortions of the harmonic temporal proportions in walking, running, and swimming (55–57).

The main limitation of this review is related to the relatively restricted number of studies available, most commonly small sample sized and with short durations. Accordingly, in many of these studies, authors failed to detect significant characteristics due to insufficient statistical power. In addition, some existing studies were not considered since the entry terms, such as for example those referring to interactive computer playing (3) or simply to serious games (58) for videogames, were not compliant with the original search.

Nonetheless, the present study provides a brief appraisal on the state of the art pointing out the most relevant aspects related to gaming technology in pediatric neurorehabilitation. This could represent a useful process seeking to raise awareness among pediatricians and other health professionals of this issue and gather their support in maximizing the use of game and technology in the clinical practice.

However, we should also consider the point of view of some therapists about gaming technologies in rehabilitation: they are often not expert in using these technologies, as well as in taking into account related privacy issues, and they questioned their role in the context of technology-based interventions and also the transferability of digital training results in real life (59).

The possibilities of gaming technologies reported in the analyzed studies included the amelioration of health service, patients' engagement, and functional outcomes, at gyms as well as at home, combined or not with other technologies. Furthermore, gaming technology allows for connecting people each other, promoting social interactions with peers and family members and avoiding isolation, while giving, at the same time, practice opportunities to children during non-therapy specific time and overall to cure and care unavoidable psychological and social aspects of chronic disabling conditions.

There are increasing experimental evidence that virtual technologies may promote also artistic interventions (5) that might be beneficial particularly to certain populations of patients (i.e., children with RDs) for rehabilitative purposes even in remote healthcare solutions as in Telemedicine (60, 61).

However, solid scientific evidences are still lacking. As highlighted by our review, further research on technological gaming is needed to provide evidence of their effects on rehabilitation: our review highlighted that studies should be based upon clearer neuroscientific principles, tested on more longitudinal randomized controlled trials to tailor games to different patient populations and various conditions, assessing technologies for accessibility, costs and acceptability. Results should be evaluated by mean of valid and reliable outcome measures, allowing for comparisons to conventional rehabilitation methods. Dedicated educational programs should also be planned to let therapists be able to manage gaming technologies with rehabilitation purposes. Lastly, research, public health, stakeholders, policymakers, health plan managers should invest on advanced telecommunications and computer technologies in order to encourage its application from in-person to remote site, especially for people living far away from health care centers.

## Author Contributions

MI searched the literature, analyzed the results supported by AEG and drafted the first version of manuscript. CMV developed the theoretical framework. MR re-drafted the further versions of manuscript. AP contributed to the design and implementation of the research, re-drafted, and revised all the versions of manuscript. All authors provided critical feedback and contributed to the final version of manuscript, read and approved the final manuscript, and conceived the original idea of the manuscript.

## Funding

This study was supported by the University of Catania – University Research Funds – Research Plan 2016/2018.

## Conflict of Interest

The authors declare that the research was conducted in the absence of any commercial or financial relationships that could be construed as a potential conflict of interest. The handling editor declared a shared affiliation with one of the authors MI at the time of review.

## Publisher's Note

All claims expressed in this article are solely those of the authors and do not necessarily represent those of their affiliated organizations, or those of the publisher, the editors and the reviewers. Any product that may be evaluated in this article, or claim that may be made by its manufacturer, is not guaranteed or endorsed by the publisher.

## References

[1] Platz T Sandrini G. Specialty grand challenge for NeuroRehabilitation research. Front Neurol. (2020) 11:349. 10.3389/fneur.2020.00349 32528395 PMC7257490

[2] Jurdi S Montaner J Garcia-Sanjuan F Jaen J Nacher V. A systematic review of game technologies for pediatric patients. Comput Biol Med. (2018) 97:89–112. 10.1016/j.compbiomed.2018.04.019 29715597

[3] Pin TW. Effectiveness of interactive computer play on balance and postural control for children with cerebral palsy: A systematic review. Gait Posture. (2019) 73:126–39. 10.1016/j.gaitpost.2019.07.122 31323621

[4] Morone G Spitoni GF De Bartolo D Ghanbari Ghooshchy S Di Iulio F Paolucci S. Rehabilitative devices for a top-down approach. Expert Rev Med Devices. (2019) 16:187–95. 10.1080/17434440.2019.1574567 30677307

[5] Iosa M Aydin M Candelise C Coda N Morone G Antonucci G. The michelangelo effect: art improves the performance in a virtual reality task developed for upper limb neurorehabilitation. Front Psychol. (2021) 11:611956. 10.3389/fpsyg.2020.611956 33488478 PMC7817887

[6] Liberati A Altman DG Tetzlaff J Mulrow C Gotzsche PC Ioannidis JP. The PRISMA statement for reporting systematic reviews and meta-analyses of studies that evaluate health care interventions: explanation and elaboration. PLoS Med. (2009) 6:e1000100. 10.1371/journal.pmed.1000100 19621070 PMC2707010

[7] de Morton NA. The PEDro scale is a valid measure of the methodological quality of clinical trials: a demographic study. Aust J Physiother. (2009) 55:129–33. 10.1016/S0004-9514(09)70043-1 19463084

[8] Luna-Oliva L Ortiz-Gutierrez RM Cano-de la Cuerda R Piedrola RM Alguacil-Diego IM Sanchez-Camarero C. Kinect Xbox 360 as a therapeutic modality for children with cerebral palsy in a school environment: a preliminary study. NeuroRehabilitation. (2013) 33:513–21. 10.3233/NRE-131001 24018364

[9] Schatton C Synofzik M Fleszar Z Giese MA Schols L Ilg W. Individualized exergame training improves postural control in advanced degenerative spinocerebellar ataxia: A rater-blinded, intra-individually controlled trial. Parkinsonism Relat Disord. (2017) 39:80–4. 10.1016/j.parkreldis.2017.03.016 28365204

[10] Do JH Yoo EY Jung MY Park HY. The effects of virtual reality-based bilateral arm training on hemiplegic children's upper limb motor skills. NeuroRehabilitation. (2016) 38:115–27. 10.3233/NRE-161302 26923353

[11] Olivieri I Meriggi P Fedeli C Brazzoli E Castagna A Roidi MLR. Computer Assisted REhabilitation (CARE) Lab: A novel approach towards Pediatric Rehabilitation 2.0. J Pediatr Rehabil Med. (2018) 11:43–51. 10.3233/PRM-160436 29630562 PMC6027946

[12] Daoud MI Alhusseini A Ali MZ Alazrai R. A game-based rehabilitation system for upper-limb cerebral palsy: a feasibility study. Sensors. (2020) 20:10.3390/s20082416. 10.3390/s20082416 32344557 PMC7219503

[13] Kommalapati R Michmizos KP. Virtual reality for pediatric neuro-rehabilitation: adaptive visual feedback of movement to engage the mirror neuron system. Annu Int Conf IEEE Eng Med Biol Soc. (2016) 2016:5849–52. 10.1109/EMBC.2016.7592058 28269584

[14] Bortone I Leonardis D Solazzi M Procopio C Crecchi A Briscese L. Serious game and wearable haptic devices for neuro motor rehabilitation of children with cerebral palsy. In: Ibáñez J González-Vargas J Azorín JM Akay M Pons JL editors. Converging Clinical and Engineering Research on Neurorehabilitation II. Biosystems & Biorobotics. Berlin: Springer (2017). 10.1007/978-3-319-46669-9_74

[15] Bortone I Leonardis D Mastronicola N Crecchi A Bonfiglio L Procopio C. Wearable haptics and immersive virtual reality rehabilitation training in children with neuromotor impairments. IEEE Trans Neural Syst Rehabil Eng. (2018) 26:1469–78. 10.1109/TNSRE.2018.2846814 29985156

[16] van Hedel HJ Hafliger N Gerber CN. Quantifying selective elbow movements during an exergame in children with neurological disorders: a pilot study. J Neuroeng Rehabil. (2016) 13:93. 10.1186/s12984-016-0200-3 27769301 PMC5073824

[17] Grossard C Hun S Serret O Grynszpan O Foulon P Dapogny A. The reeducation of emotional expressions for children with autism spectrum disorders thanks to information communication technologies: JEMImE project. Neuropsychiatrie de l'Enfance et de l'Adolescence. (2017) 65:21–32. 10.1016/j.neurenf.2016.12.002

[18] Pedroli E Padula P Guala A Meardi MT Riva G Albani G. A psychometric tool for a virtual reality rehabilitation approach for dyslexia. Comput Math Methods Med. (2017) 2017:7048676. 10.1155/2017/7048676 28286543 PMC5327757

[19] Summa S Schirinzi T Bernava GM Romano A Favetta M Valente EM. Development of SaraHome: A novel, well-accepted, technology-based assessment tool for patients with ataxia. Comput Methods Programs Biomed. (2020) 188:105257. 10.1016/j.cmpb.2019.105257 31846831

[20] Wu YN Saliu V Donoghue ND Donoghue JP Kerman KL. A home-based massed practice system for pediatric neurorehabilitation. In: Pons J Torricelli D Pajaro M editors. Converging Clinical and Engineering Research on Neurorehabilitation. Biosystems & Biorobotics, vol 1. Biosystems & Biorobotics, vol 1. ed. Berlin: Springer. (2013) p. 1003–7. 10.1007/978-3-642-34546-3_164

[21] Reifenberg G Gabrosek G Tanner K Harpster K Proffitt R Persch A. Feasibility of pediatric game-based neurorehabilitation using telehealth technologies: A case report. Am J Occup Ther. (2017) 71:24976. 10.5014/ajot.2017.024976 28422630

[22] Gerber CN Kunz B van Hedel HJ. Preparing a neuropediatric upper limb exergame rehabilitation system for home-use: a feasibility study. J Neuroeng Rehabil. (2016) 13:33. 10.1186/s12984-016-0141-x 27008504 PMC4806437

[23] Kang YS Chang YJ. Using game technology to teach six elementary school children with autism to take a shower independently. Dev Neurorehabil. (2019) 22:329–37. 10.1080/17518423.2018.1501778 30060690

[24] Valdes BA Glegg SMN Lambert-Shirzad N Schneider AN Marr J Bernard R. Application of commercial games for home-based rehabilitation for people with hemiparesis: challenges and lessons learned. Games Health J. (2018) 7:197–207. 10.1089/g4h.2017.0137 29565694

[25] Dehem S Montedoro V Brouwers I Edwards MG Detrembleur C Stoquart G. Validation of a robot serious game assessment protocol for upper limb motor impairment in children with cerebral palsy. NeuroRehabilitation. (2019) 45:137–49. 10.3233/NRE-192745 31498135

[26] Keller U van Hedel HJA Klamroth-Marganska V Riener R. ChARMin: The first actuated exoskeleton robot for pediatric arm rehabilitation. EEE/ASME Transactions on Mechatronics. (2021) 21:2201–13. 10.1109/TMECH.2016.255979927295638

[27] Keller JW van Hedel HJA. Weight-supported training of the upper extremity in children with cerebral palsy: a motor learning study. J Neuroeng Rehabil. (2017) 14:87. 10.1186/s12984-017-0293-3 28854939 PMC5577664

[28] Ricklin S Meyer-Heim A van Hedel HJA. Dual-task training of children with neuromotor disorders during robot-assisted gait therapy: prerequisites of patients and influence on leg muscle activity. J Neuroeng Rehabil. (2018) 15:82. 10.1186/s12984-018-0426-3 30223840 PMC6142352

[29] Labruyere R Gerber CN Birrer-Brutsch K Meyer-Heim A van Hedel HJ. Requirements for and impact of a serious game for neuro-pediatric robot-assisted gait training. Res Dev Disabil. (2013) 34:3906–15. 10.1016/j.ridd.2013.07.031 24025439

[30] Brutsch K Schuler T Koenig A Zimmerli L-Koeneke SM Lunenburger L. Influence of virtual reality soccer game on walking performance in robotic assisted gait training for children. J Neuroeng Rehabil. (2010) 7:15. 10.1186/1743-0003-7-15 20412572 PMC2877051

[31] Giannopulu I Pradel G. Multimodal interactions in free game play of children with autism and a mobile toy robot. NeuroRehabilitation. (2010) 27:305–11. 10.3233/NRE-2010-0613 21160119

[32] Giannopulu I. Multimodal cognitive nonverbal and verbal interactions: The neurorehabilitation of autistic children via mobile toy robots. Int J Adv Life Sci. (2013) 5:214–22.

[33] Calderita LV Manso LJ Bustos P Suarez-Mejias C Fernandez F Bandera A. THERAPIST: Towards an autonomous socially interactive robot for motor and neurorehabilitation therapies for children. JMIR Rehabil Assist Technol. (2014) 1:e1. 10.2196/rehab.3151 28582242 PMC5454569

[34] Bortone I Barsotti M Leonardis D Crecchi A Tozzini A Bonfiglio L. Immersive virtual environments and wearable haptic devices in rehabilitation of children with neuromotor impairments: a single-blind randomized controlled crossover pilot study. J Neuroeng Rehabil. (2020) 17:144. 10.1186/s12984-020-00771-6 33115487 PMC7594483

[35] Choi JY Yi SH Ao L Tang X Xu X Shim D. Virtual reality rehabilitation in children with brain injury: a randomized controlled trial. Dev Med Child Neurol. (2021) 63:480–7. 10.1111/dmcn.14762 33326122

[36] Decavele S Ortibus E Van Campenhout A Molenaers G Jansen B Omelina L. The effect of a rehabilitation specific gaming software platform to achieve individual physiotherapy goals in children with severe spastic cerebral palsy: a randomized crossover trial. Games Health J. (2020) 376–85. 10.1089/g4h.2019.0097 32614723

[37] Golomb MR Warden SJ Fess E Rabin B Yonkman J Shirley B. Maintained hand function and forearm bone health 14 months after an in-home virtual-reality videogame hand telerehabilitation intervention in an adolescent with hemiplegic cerebral palsy. J Child Neurol. (2011) 26:389–93. 10.1177/0883073810394847 21383228 PMC4290160

[38] Gorelik V Filippova SN Belyaev VS Karlova EV. Efficiency of image visualization simulator technology for physical rehabilitation of children with cerebral palsy through play. Bull RSMU. (2019) 4:39–46. 10.24075/brsmu.2019.051

[39] Tarakci D Ersoz Huseyinsinoglu B Tarakci E Razak Ozdincler A. Effects of Nintendo Wii-Fit((R)) video games on balance in children with mild cerebral palsy. Pediatr Int. (2016) 58:1042–50. 10.1111/ped.12942 26858013

[40] Zoccolillo L Morelli D Cincotti F Muzzioli L Gobbetti T Paolucci S. Video-game based therapy performed by children with cerebral palsy: a cross-over randomized controlled trial and a cross-sectional quantitative measure of physical activity. Eur J Phys Rehabil Med. (2015) 51:669–76. 25653079

[41] Pagliano E Foscan M Marchi A Corlatti A Aprile G Riva D. Intensive strength and balance training with the Kinect console (Xbox 360) in a patient with CMT1A. Dev Neurorehabil. (2018) 21:542–5. 10.1080/17518423.2017.1354091 28762860

[42] de Paula JN de Mello Monteiro CB da Silva TD Capelini CM de Menezes LDC Massetti T. Motor performance of individuals with cerebral palsy in a virtual game using a mobile phone. Disabil Rehabil Assist Technol. (2018) 13:609–13. 10.1080/17483107.2017.1392620 29092683

[43] Ravi DK Kumar N Singhi P. Effectiveness of virtual reality rehabilitation for children and adolescents with cerebral palsy: an updated evidence-based systematic review. Physiotherapy. (2017) 103:245–58. 10.1016/j.physio.2016.08.004 28109566

[44] Wu J Loprinzi PD Ren Z. The rehabilitative effects of virtual reality games on balance performance among children with cerebral palsy: a meta-analysis of randomized controlled trials. Int J Environ Res Public Health. (2019) 16:6214161. 10.3390/ijerph16214161 31661938 PMC6861947

[45] Bonnechere B Jansen B Omelina L Degelaen M Wermenbol V Rooze M. Can serious games be incorporated with conventional treatment of children with cerebral palsy? A review. Res Dev Disabil. (2014) 35:1899–913. 10.1016/j.ridd.2014.04.016 24794289

[46] Deutsch JE Guarrera-Bowlby P Myslinski MJ Kafri M. Is there evidence that active videogames increase energy expenditure and exercise intensity for people poststroke and with cerebral palsy? Games Health J. (2015) 4:31–7. 10.1089/g4h.2014.0082 26181678

[47] Lai B Lee E Kim Y Matthews C Swanson-Kimani E Davis D. Leisure-time physical activity interventions for children and adults with cerebral palsy: a scoping review. Dev Med Child Neurol. (2021) 63:162–71. 10.1111/dmcn.14751 33241561

[48] Coco-Martin MB Pinero DP Leal-Vega L Hernandez-Rodriguez CJ Adiego J Molina-Martin A. The potential of virtual reality for inducing neuroplasticity in children with amblyopia. J Ophthalmol. (2020) 2020:7067846. 10.1155/2020/7067846 32676202 PMC7341422

[49] Janssen J Verschuren O Renger WJ Ermers J Ketelaar M van Ee R. Gamification in physical therapy: more than using games. Pediatr Phys Ther. (2017) 29:95–9. 10.1097/PEP.0000000000000326 27984481

[50] Grossman SN Han SC Balcer LJ Kurzweil A Weinberg H Galetta SL. Rapid implementation of virtual neurology in response to the COVID-19 pandemic. Neurology. (2020) 94:1077–87. 10.1212/WNL.0000000000009677 32358217

[51] Rubinstein YR Robinson PN Gahl WA Avillach P Baynam G Cederroth H. The case for open science: rare diseases. JAMIA Open. (2020) 3:472–86. 10.1093/jamiaopen/ooaa030 33426479 PMC7660964

[52] Ruggieri M Polizzi A Marceca GP Catanzaro S Pratico AD Di Rocco C. Introduction to phacomatoses (neurocutaneous disorders) in childhood. Childs Nerv Syst. (2020) 36:2229–68. 10.1007/s00381-020-04758-5 32940773

[53] Chessa L Ruggieri M Polizzi A. Progress and prospects for treating ataxia telangiectasia. Expert Opinion Orphan Drugs. (2021) 7:233–51. 10.1080/21678707.2019.1623022

[54] Ruggieri M Pratico AD Evans DG. Diagnosis, management, and new therapeutic options in childhood neurofibromatosis type 2 and related forms. Semin Pediatr Neurol. (2015) 22:240–58. 10.1016/j.spen.2015.10.008 26706012

[55] Ricci M Terribili M Giannini F Errico V Pallotti A Galasso C. Wearable-based electronics to objectively support diagnosis of motor impairments in school-aged children. J Biomech. (2019) 83:243–52. 10.1016/j.jbiomech.2018.12.005 30554812

[56] Verrelli CM Iosa M Roselli P Pisani A Giannini F Saggio G. Generalized finite-length fibonacci sequences in healthy and pathological human walking: comprehensively assessing recursivity, asymmetry, consistency, self-similarity, and variability of gaits. Front Hum Neurosci. (2021) 15:649533. 10.3389/fnhum.2021.649533 34434095 PMC8381873

[57] Verrelli CM Romagnoli C Jackson RR Ferretti I Annino G Bonaiuto V. Front crawl stroke in swimming: Phase durations and self-similarity. J Biomech. (2021) 118:110267. 10.1016/j.jbiomech.2021.110267 33571818

[58] Velasco MA Raya R Muzzioli L Morelli D Otero A Iosa M. Evaluation of cervical posture improvement of children with cerebral palsy after physical therapy based on head movements and serious games. Biomed Eng Online. (2017) 16(Suppl 1):74. 10.1186/s12938-017-0364-5 28830552 PMC5568605

[59] Tatla SK Shirzad N Lohse KR Virji-Babul N Hoens AM Holsti L. Therapists' perceptions of social media and video game technologies in upper limb rehabilitation. JMIR Serious Games. (2015) 3:e2. 10.2196/games.3401 25759148 PMC4373832

[60] Polizzi A Gentile AE Taruscio D. Competing to raise awareness of rare diseases. Lancet Neurol. (2019) 18:721–2. 10.1016/S1474-4422(18)30437-X 30447970

[61] Iosa M Gentile AE Verrelli CM Ruggieri M Polizzi A. Telemedicine and health humanities for children with rare diseases: a lesson from COVID-19 to e-Psychology. EC Psychol Psychiatry. (2021) 10:1−3.

